# Differences in facial expressions during positive anticipation and frustration in dogs awaiting a reward

**DOI:** 10.1038/s41598-019-55714-6

**Published:** 2019-12-17

**Authors:** Annika Bremhorst, Nicole A. Sutter, Hanno Würbel, Daniel S. Mills, Stefanie Riemer

**Affiliations:** 1 Division of Animal Welfare, DCR-VPHI, Vetsuisse Faculty, University of Bern, 3012 Bern, Switzerland; 2 Graduate School for Cellular and Biomedical Sciences (GCB), University of Bern, 3012 Bern, Switzerland; 3 School of Life Sciences, University of Lincoln, Lincoln, LN6 7DL United Kingdom

**Keywords:** Behavioural methods, Animal behaviour

## Abstract

Facial expressions are considered sensitive indicators of emotional states in humans and many animals. Identifying facial indicators of emotion is a major challenge and little systematic research has been done in non-primate species. In dogs, such research is important not only to address fundamental and applied scientific questions but also for practical reasons, since many problem behaviours are assumed to have an emotional basis, e.g. aggression based on frustration. Frustration responses can occur in superficially similar contexts as the emotional state of positive anticipation. For instance, the anticipated delivery of a food reward may induce the state of positive anticipation, but over time, if the food is not delivered, this will be replaced by frustration. We examined dogs’ facial expressions in contexts presumed to induce both positive anticipation and frustration, respectively, within a single controlled experimental setting. Using DogFACS, an anatomically-based method for coding facial expressions of dogs, we found that the “Ears adductor” action was more common in the positive condition and “Blink”, “Lips part”, “Jaw drop”, “Nose lick”, and “Ears flattener” were more common in the negative condition. This study demonstrates how differences in facial expression in emotionally ambiguous contexts may be used to help infer emotional states of different valence.

## Introduction

Emotional reactions are short-lasting affective responses^1^ to rewarding and punishing events^2^. Emotions can be classified either within a multidimensional space, often along two axes comprising arousal (i.e. high versus low) and hedonic valence (i.e. positivity versus negativity of an emotion; e.g.^3^), or as discrete states (e.g. happiness, fear, frustration; e.g.^4^). Both approaches are applied in non-human animal research, and they are not necessarily mutually exclusive but can be integrated (e.g. localizing discrete emotional states within the valence and arousal dimensions)^2^. To draw inferences about the emotional states of animals, we need to identify measurable proxy indicators^5^. Established indicators of emotional states enable us to answer fundamental proximate and ultimate research questions, such as how different emotions are expressed in different species or whether commonalities can be observed between them (see e.g.^6^), as well as to address questions in the applied sciences.

Behaviour is used as a marker of several components of emotions^7^ and so can be a valuable for inferring the emotional states of an animal. Alongside alterations in physiological and cognitive processes^2,7^, emotions are accompanied by changes in an individual’s behavioural expression^2,7,8^, including changes in motor action patterns, body postures, and facial expressions^9^. Human emotion research has focused extensively on facial expressions to help identify predictive indicators of emotional states (see, for example^10–14^). Facial movements are also present in most mammalian species^15,16^ and are assumed to convey information about emotional states in non-human animals as well (see for a review^5,17^). Consequently, facial expressions offer considerable potential as indicators of emotional states in animals^5^, and they are receiving increasing attention in research on animal emotions (see e.g.^18–22^).

In human emotion research, the Facial Action Coding System (FACS)^23,24^ is widely considered the gold standard for measuring facial emotional expressions^6,25^. FACS is anatomically-based, systematically describing facial appearance changes based on movements of the underlying facial musculature, hence, facilitating objective and standardised measurements of facial expression^26^. Thus, rather than considering facial expressions holistically (e.g. identifying a face as “happy” or a particular expression as a “smile”, without specifying the relevant facial features in more detail), FACS decomposes and objectively describes distinct facial features^6^. FACS requires training and a certification by the coder in order to be used reliably in a scientific context (e.g.^26–28^). Having originally been developed for humans, FACS has more recently been adapted for different non-human species including dogs (DogFACS^26^). Thus, there is now the potential to use Facial Action Coding Systems for the investigation of emotions in selected non-human animal species as well.

The domestic dog is a species where research interest on emotional states has been increasing in recent years (see for a review^29^). Dogs are morphologically diverse, highly social domestic animals who are closely integrated into human social networks^30^, and the human environment can be considered the natural ecological niche of this species^31^. This close cohabitation requires safe interactions between humans and dogs, for which we need to interpret dogs’ behavioural expressions correctly^32^. Dogs have been shown to produce different facial configurations in different emotional states (e.g.^6^), and it has even been suggested that dogs may have evolved certain facial expressions as a result of domestication that are specifically attractive to humans^26,33^. Thus, the dog provides a unique non-primate model for studying emotional expressions that are of interest to humans.

Research investigating emotional expressions in dogs has investigated affective vocalisations (e.g^34^), body expressions, and general behavioural responses of dogs in different emotional states (e.g.^35–40^; see^41^ for a review on dog communication). Furthermore, dogs’ faces appear to convey important information of communicative value concerning their emotional state (e.g.^6,42^). For instance, increased occurrence of mouth-licking behaviour, originally described in relation to acute stress in dogs^43^, has been found to occur more precisely when dogs were confronted with images depicting a negative human facial expression, and not so much when presented with a negative dog facial expression^42^. However, this study^42^ did not provide a precise definition on the mouth-licking behaviour, of which there are several forms, for example varying in the extent of tongue protrusion (only just protruding from the mouth, wiping the lips, or licking up to the nose) or possibly lateralised effects which might have different communicative significance. DogFACS^26^ provides a means to objectively code such subtlety and variation.

DogFACS^26^ has recently been used to compare differences between the facial expressions of a sample of dogs and their closest extant relatives, gray wolves^33^. This indicated a potential difference in a specific facial expression (the inner brow raiser) that led the authors to suggest that this difference evolved during domestication specifically for interspecies communication with humans^33^. DogFACS^26^ has also been used to identify that the performance of the inner eye brow raiser may affect the selection of shelter dogs by humans^26^. More broadly, DogFACS may enable quantification of both the type and amount of facial activity in different contexts, e.g. in relation to human attention and/or by an arousing stimulus (food)^44^. This has been used to argue that dogs tailor their facial expressions according to their potential audience, providing evidence of their social communicative function^44^. With regard to emotional states, DogFACS has recently been applied to assess the facial expressions of dogs in videos relating to four different putative emotional states defined by context: positive anticipation, happiness, fear, and frustration^6^.

Frustration is an aversive emotional state^36,45^ that can arise in a range of situations (see^46^), such as when an expected reward is absent, delayed^45^, reduced in value (reviewed in^47^), or inaccessible due to barriers^4^, which can be of a physical or social nature. Frustration is closely linked to the emotional state of positive anticipation, which is assumed to arise when a reward is expected^48–50^. However, if access to the expected reward is denied, positive anticipation may eventually turn into frustration; consequently, both emotional states can arise in similar circumstances – such as the withholding of food. Anticipatory behavioural expressions are often observed prior to an animal obtaining food^49^, and this primary reinforcer has been used with different species to study behavioural responses when expecting a food reward or when this expectation is thwarted (e.g.^36,48,51–55^).

Behavioural expressions of positive anticipation seem to be, at least in part, species-specific. In some species, anticipation of a positive event is associated with an increase in activity (e.g. rats^48,56^; pigs^57^; foxes^58^; horses^52^), whereas in other species it is associated with a reduction in activity (e.g. cats^48^; fowl^59^). In dogs, one study has shown that their behavioural response when anticipating a reward was dependent on the animals’ ability to control access to the desired stimulus^38^. When the reward was accessible by performing an operant behaviour on a previously trained device, the dogs showed a higher activity level and frequency of tail wagging compared to control dogs who were not previously trained on the device and, hence, could not control access to the reward^38^. Control dogs also showed biting and chewing behaviours towards the operant device which was not observed for any trained dog, and were reluctant to enter the test area after the first few trials^38^. These observations are consistent with the control dogs experiencing a negative state akin to frustration. However, signs of frustration may not always be obvious and behavioural changes can be hard to interpret (c.f.^60^). In two other frustration-related studies, dogs showed several behaviour changes such as lying down and increasing their distance from the experimenter who had previously been rewarding them^36,54^. The dogs also showed increasing ambulation, vocalization and sniffing at this time^36^. By contrast, in the frustration-provoking situation of another study, with an experimenter withholding food by keeping a treat in her closed hand, dogs manipulated the hand with their mouth, stood motionless, and gazed at the experimenter^55^. Thus, the overt behavioural tendencies of dogs to frustration might be quite variable, possibly depending on the specifically frustrating context. Indeed in the latter study^55^, the dogs also showed an increase in nose and lip licking, which may relate to overt communicative signals associated with the conflict related to frustration around humans or the specific use of food in this context.

Caeiro and colleagues (2017)^6^ were the first to specifically investigate facial expressions of dogs during positive anticipation and frustration (as well as during happiness and fear) using DogFACS^26^. In this study^6^, the spontaneous response of dogs of different breeds and mixes was assessed using online videos depicting contexts that were associated with the target emotional states. Initially, relevant contextual criteria and triggering stimuli associated with each emotional state were defined. Positive anticipation was defined as being induced by the “[v]isualisation of food or hearing meal/food related word(s); [v]isualisation of leash, hearing walk related word(s).”^6^. The dogs’ facial expressions were then measured using DogFACS^26^ from the point of stimulus presentation until the consummatory phase of the behaviour commenced^6^. Frustration was defined as being induced by the “[v]isualisation of a desired resource (toy, food, space) that is or becomes inaccessible.”^6^. Dogs’ facial expressions were then measured from the point when the subject attempted to access the resource for the first time and during its subsequent denials^6^. The authors found that positive anticipation was characterized by a higher rate of “Lip wipe” (DogFACS^26^ Action Descriptor 37 = AD37; i.e. the dogs wiping their lips with the tongue, see^26^, www.dogfacs.com) or “Nose lick” (AD137) and “Ears adductor” (DogFACS^26^ Ear Action Descriptor 102 = EAD102; i.e. the ears move towards the midline of the head making the ear bases coming closer together, see^26^, www.dogfacs.com) relative to the control phase in which emotion-inducing stimuli were absent^6^. However, the authors could not identify distinguishing facial indicators of frustration relative to their baseline.

Therefore, the aim of the current study was to investigate dogs’ facial expressions of frustration and positive anticipation using DogFACS, in a controlled experimental setting unlike Caeiro *et al*. (2017)^6^. Furthermore, in order to maximize the potential to detect possible signals of importance, we standardised the dog breed (Labrador Retriever) to reduce the potential effects of morphological variation and extremes on the dogs’ facial expressions; we also used a non-social context in order to eliminate the risk of interference from previously learned attention getting responses. A high-value food reward was used as the triggering stimulus in two conditions – a positive condition predicted to induce positive anticipation and a negative condition predicted to induce frustration in dogs (Table 1).

**Table 1:** Condition, presumed valence, assumed emotional state, definition of the emotional state, trigger, and experimental contexts used in the present study.

Condition	Presumed valence	Assumed emotional state	Definition	Trigger	Experimental contexts
Positive	Positive	Positive anticipation	Emotion induced when access to a reward is expected;^48,49^ time interval between signal and arrival of a reward^50^.	Food	Expectation of access to a high-value food reward.
Negative	Negative	Frustration	Emotion induced through absence, delay or inaccessibility (through social or physical barriers) of an expected reward^4,45^.		Denial of access to a high-value food reward, which is visible but not accessible through a perspex panel.

During pre-training, dogs learned to approach an apparatus from which a food reward was delivered after five seconds and could immediately be consumed. This procedure was also used in the subsequent testing trials (N = 15) to induce positive anticipation. In randomly interspersed trials of the negative condition (N = 5), the subjects could see but not access the food for up to 55 seconds. For each subject, video samples from repeated trials of both conditions were analysed by a blinded certified coder using DogFACS^26^. Based on the presence or absence of selected DogFACS variables in the two conditions, we analysed whether facial expressions differed between the two conditions. *A priori* hypotheses relating to potential DogFACS^26^ variables of interest were not specified, as none have been identified previously.

## Results

Eleven DogFACS variables were used for the analysis, based on a prevalence of at least 10% across all samples of either the positive or negative condition and at least a substantial strength of intercoder agreement^61^ (see Supplementary Table 1).

First, within conditions, binomial logistic regression models were used to test whether there was an effect of sample across the repeated samples within the positive and the negative condition, respectively. Models could be computed for all but three variables due to zero inflation: “Nose lick” (AD137) and “Panting” (AD126; both rare during the positive condition), and “Ears adductor” (EAD102; rare during the negative condition). For all other variables, results indicated that sample did not contribute significant information to explaining our data (Table 2). Therefore, we pooled the data of each condition to assess the effect of condition (positive/ negative) on the occurrence of the eleven DogFACS variables.

**Table 2:** Within condition analyses. Results of the binomial logistic regression models comparing multiple samples within the positive and negative condition, respectively, for the eleven DogFACS variables (#: model calculation not possible due to zero inflation).

DogFACS variable	POSITIVE condition			NEGATIVE condition		
	χ^2^	df	P (Holm-Bonferroni corrected)	χ^2^	df	P (Holm-Bonferroni corrected)
1. Inner brow raiser (AU101)	1.82	2	0.402 (1)	1.09	5	0.955 (1)
2. Blink (AU145)	0.41	2	0.814 (1)	5.90	5	0.316 (1)
3. Lip corner puller (AU12)	0.70	2	0.703 (1)	4.03	5	0.546 (1)
4. Lower lip depressor (AU116)	2.62	2	0.270 (1)	4.11	5	0.534 (1)
5. Lips part (AU25)	1.77	2	0.414 (1)	3.32	5	0.651 (1)
6. Jaw drop (AU26)	0.53	2	0.768 (1)	5.11	5	0.403 (1)
7. Tongue show (AD19)	0.17	2	0.921 (1)	1.85	5	0.869 (1)
8. Nose lick (AD137)	#	#	#	0.87	5	0.972 (1)
9. Ears adductor (EAD102)	1.72	2	0.423 (1)	#	#	#
10. Ears flattener (EAD103)	2.20	2	0.334 (1)	3.64	5	0.603 (1)
11. Panting (AD126)	#	#	#	7.75	5	0.171 (1)

**Figure 1:**
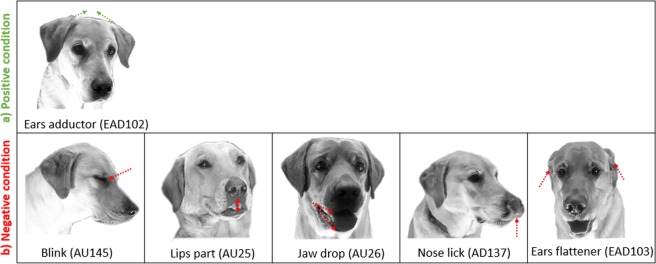
DogFACS variables more common in (**a**) the positive and (**b**) the negative condition.

Between the two conditions, binomial logistic regression models indicated that six of the eleven DogFACS variables differed between the positive and the negative condition, based on the 95% confidence intervals not including zero (Table 3, Fig. 1). “Ears adductor” (EAD102) was the only variable that was more common in the positive condition while “Blink” (Action Unit 145 = AU145), “Lips part” (AU25), “Jaw drop” (AU26), “Nose lick” (AD137), and “Ears flattener” (EAD103) were more common in the negative condition.

**Table 3:** Between condition analyses. Results of the binomial logistic regression models comparing the pooled data of the positive and negative condition for the eleven DogFACS variables.

DogFACS variable	χ^2^	df	Estimate	SE	z	R^2^	CI		P (Holm-Bonferroni corrected)
							2.5%	97.5%	
1. Inner brow raiser (AU101)	0.13	1	−0.13	0.36	−0.35	0.19	−0.86	0.57	0.723 (1.000)
**2.Blink (AU145)**	6.91	1	0.85	0.32	2.63	0.18	0.23	1.51	0.009 (0.051)
3. Lip corner puller (AU12)	1.82	1	0.67	0.50	1.35	0.52	−0.28	1.69	0.177 (0.797)
4. Lower lip depressor (AU116)	1.98	1	−0.70	0.50	−1.41	0.30	−1.70	0.28	0.159 (0.797)
**5. Lips part (AU25)**	10.61	1	1.60	0.49	3.26	0.63	0.69	2.64	0.001 (0.01)
**6. Jaw drop (AU26)**	12.58	1	1.68	0.47	3.55	0.59	0.80	2.67	<0.001(0.004)
7. Tongue show (AD19)	1.28	1	0.61	0.54	1.13	0.65	−0.42	1.72	0.257 (0.797)
**8. Nose lick (AD137)**	9.50	1	2.37	0.77	3.08	0.24	1.08	4.24	0.002 (0.014)
**9. Ears adductor (EAD102)**	10.34	1	−1.79	0.56	−3.22	0.08	−2.97	−0.75	0.001 (0.01)
**10. Ears flattener (EAD103)**	29.46	1	1.84	0.34	5.43	0.34	1.20	2.54	<0.001 (<0.001)
11. Panting (AD126)	0.43	1	0.32	0.49	0.65	0.53	−0.63	1.31	0.513 (1.000)

Variables with bold letters differed between the two conditions, based on the 95% confidence interval not containing zero.

## Discussion

Using a within-subject design, this study explored facial expressions of dogs in two superficially similar conditions involving a food reward: (1.) positive: expectation of access to a high-value food reward (i.e. positive anticipation), and (2.) negative: denial of access to the food reward (i.e. frustration). As no validated measures of positive anticipation and frustration in dogs are available yet, the assumption of the underlying emotional state is based on criteria provided by the literature (e.g.^4,6,45,46,48,49^), specifying contextual features of situations in which the putative states of positive anticipation and frustration are induced. Two ear actions were found to differ between the two conditions, with activity in the “Ears adductor” (EAD102) being more common in the positive and “Ears flattener” (EAD103) in the negative condition. Furthermore, one eye action (“Blink”: AU145) and several mouth actions (“Jaw drop”: AU26, “Lips part”: AU25, “Nose lick”: AD137) were also more common in the negative condition. The findings demonstrate for the first time that the two contrary states of positive anticipation and frustration are associated with different facial expressions in dogs.

The association of the “Ears adductor” (EAD102) action with the putative state of positive anticipation replicates the result of Caeiro and colleagues (2017)^6^, enhancing the validity of the finding. Aural signals appear to be used often as part of the emotional display in animals^6^, but the affective semiotic content of ear postures and movements varies across species. Like dogs, silver foxes have more erect ears (resembling the action of the DogFACS “Ears adductor”: EAD102), when anticipating a positive reward^58^, and cats too have been shown to increase “Ears adductor” activity in positive situations, i.e. when the animals were engaging with the environment in a relaxed manner^62^. In sheep, however, a movement akin to the “Ears adductor” (EAD102) action, has been linked to negative emotional states^63^. Besides being considered as an indicator of emotional states, ear movement has also been discussed as a cue of attentional state in animals with mobile ears^64,65^.

In contrast to Caeiro and colleagues (2017)^6^, who did not find specific facial features expressed by dogs during the putative state of frustration compared to baseline, the present study indicates that at least within the context of food being withheld, distinct facial expressions were associated with frustration compared to positive anticipation. This involved actions of the ears, mouth, and eye region. The “Ears flattener” (EAD103) occurred more often during the putative state of frustration. This caudal turning of the ears has been associated with negative emotional states in a range of other species (e.g. sheep^63^; goats^66^; pigs^67^; horses^68,69^: see^70^; cats^62^; but see cows^19^). In canids, including the coyote, fox, and wolf, flattened ears are also observed in frightening situations^71^. In dogs, flattened ears are often considered to be part of a submissive display^72–74^ and interpreted as a sign of fear^73,75^. In a study with silver foxes that were trained in a Pavlovian conditioning paradigm to associate a bell sound with either a predictable food reward (a piece of salmon), an unpredictable reward (food related: dog treats, cattle humerus, salmon; or not food related: tennis ball or wooden stick), or a negative predictable treatment (an aversive stimulus: being captured by grabbing the neck), flat and backwards rotated ears were seen when anticipating the negative predictable treatment but also the positive unpredictable reward^58^. Thus, together with our own results it seems that flattened ears are not only a sign of a fearful state, but also a frustrating one in canids. This indicates that the action of the “Ears flattener” (EAD103) may be a more general indicator of states of negative valence in dogs, rather than being associated with a distinct discrete emotion (i.e. fear or frustration). Future studies should explore the extent to which the “Ears flattener” (EAD103) action is observed in dogs across different negatively valenced situations to determine the validity of this generalization.

Future work, evaluating different measures of the “Ears flattener” (EAD103) action (i.e. duration, frequency, presence/absence, transitions of actions) and finer details of ear movement might provide deeper insight into the differentiation of the specific negative states associated with this action. In a study on emotional expressions of cows, backwards ears were subclassified into “ears back up” and “ears back down”, and these two actions were differentially expressed in different emotional states^19^. Such a subclassification may also be done with the “Ears flattener” (EAD103) action expressed by dogs. However, the feasibility of coding this ear movement might differ depending on the dogs’ natural ear shape.

Furthermore, animals with mobile ears can usually move both pinnae independently from each other, and asymmetrical ear movements have been reported in studies on emotional expressions in different animal species (e.g.^19,63,70^). Also in humans, facial asymmetry and laterality have been discussed in the context of emotional processes^76,77^. Thus, it may be of interest to measure bilateral postures and independent movements of the two ears in dogs in order to identify asymmetries (differences between the two ears^76^) and lateralities (consistent asymmetries or biases for one ear^76^) in relation to different emotional states. Lateralized behaviours such as tail wagging or gazing in different emotional situations have already been observed in dogs^35,78,79^ (see for a review^80^). For example, dogs’ tail wagging amplitude has been reported to have a bias towards the right side when facing the owner, an unfamiliar human, or a cat; whereas a left-side bias was observed when facing an unfamiliar conspecific or when being alone^35^. A bias for a head movement towards the left side was observed in dogs in response to being confronted with an alarming or threatening visual 2D stimulus (cat or snake)^79^. Whether asymmetrical ear movements provide similar information on the emotional states of dogs has yet to be determined.

As in Caeiro *et al*. (2017)^6^, in the present study most facial expressions that differed between the positive and negative condition were in the mouth area. However, there were some contradictory findings between the two studies. In addition to an increase in “Ears adductor” (EAD102) during positive anticipation (as in the current study), Caeiro and colleagues (2017)^6^ reported an increase in “Lip wipe” (AD37) and “Nose lick” (AD137). In contrast, in the present study, “Nose lick” (AD137) was more common in the negative condition. This finding might reflect differences in the context of the two studies. The earlier study^6^, while it did not use overtly aversive contexts, it did not control for reward delay to the same degree as here. Nose and lip licking are often considered to be appeasement signals in dogs that have been observed during interspecific (e.g. dog-human^81,82^) and intraspecific communication (e.g.^83^). Appeasement gestures are assumed to be displayed in potentially conflicting social situations in order to reduce arousal in the sender or others^84^. However, empirical studies testing the appeasing effect are rare (but see^81,83^). Increased frequencies of nose or lip licking have furthermore been reported in dogs in a frustration-provoking situation^55^, during more general states of increased arousal^82^, and during stressful events (e.g.^43^, but see^85^). Although research on stress and emotion has often been separated^86,87^, the two topics are associated^88^ and overlapping^89^. “When there is stress there are also emotions” (p. 35)^86^, thus, stress is more fully defined as a subset of emotional states^87^. It is perhaps most useful to consider stress as a general increase in physiological arousal/demand associated with a salient stimulus, while the emotion depends on the perceived relationship between the individual and the stimulus (e.g. desirable, a barrier to something, a threat). Oftentimes, stress responses are measured in situations that are aversive in some way, meaning harmful, threatening, or challenging^86^, and thus, very likely inducing negative emotional states^89^. In which case it is perhaps clearer to refer to this state as a form of “distress”, to distinguish it from an increase in cortisol which is simply indicative of anticipation of an increased demand, without a specific valence. Disentangling behavioural expressions of stress and emotion is challenging, since the two are not mutually exclusive; behaviours identified as indicators of a stressful state may also accompany different emotional states.

In the present study, two additional mouth behaviours differed between the positive and negative condition – “Jaw drop” (AU26) and “Lips part” (AD25) were both more common during the putative state of frustration. The behaviours are usually not mutually exclusive and are commonly shown in combination with either each other or with additional facial actions such as for instance with “Tongue show” (AD19), “Nose lick” (AD137), “Lip wipe” (AD37), or “Panting” (AD126). Although panting has been related to stressful events in dogs^43,90^, it was not more common in either state in the present study.

There were also differences in the eye area between the positive and negative condition, with “Blink” (AU145) being more common during the putative state of frustration. Variation in eyelid aperture is a common feature of many emotional displays, both during positive and negative emotional states (reviewed by^5^). Different variations of the eye area have been identified as emotional indicators, for instance eye wrinkles are associated with certain negative emotional states in horses^51^ and increased visibility of the white of the eyes is associated with frustration in cows^22^. Blinking in particular has been associated with fear in cats^62^. In dogs, blinking seems to have been considered as an appeasement signal^37,41^, although not specifically tested for this function. The present study, however, provides evidence of a specific association between blinking in dogs with a state of frustration, which may be a precondition for appeasement.

The lack of a baseline measure for every tested individual in the present study means that the significant facial actions allow us to differentiate the positive and the negative condition and not specifically characterise frustration and positive anticipation. However, the replication of the finding of Caeiro *et al*. (2017)^6^ for positive anticipation relative to their baseline, and similarity of the identified expressions during the frustration condition to more general observations of signals potentially associated with negative emotional states, stress and arousal (e.g.^6,42,43^), as discussed above, indicate that the measures we found are indeed important in the characterisation of positive anticipation and frustration, respectively. However, we agree with Caeiro and colleagues (2017)^6^ that we must also consider the possibility that expressions of frustration may have a degree of context-specificity. Certainly, in relation to the behavioural tendencies shown at this time, these can be expected to vary with their context-specific goal^46,91^ and it is possible that the facial signals may vary depending on the social target of any communication^42^. Future studies will need to systematically vary contextual features of situations thought to induce frustration and positive anticipation (e.g. by using different emotion-triggering stimuli to food such as toys) to identify common denominators across comparable contexts for each emotional state.

Since facial expressions could potentially be affected by morphological differences^6^, only one breed without morphological extremes, the Labrador Retriever, was tested in the present study. However by doing this, it might be argued that the generalizability of our results is limited. In order to assess the external validity of the present findings, it would be valuable to test a larger range of dog breeds in a future study. However, we assume that generalizability of our findings to different dog breeds and mixes is likely in view of the finding^6^ that there does not appear to be any significant differences in the production of selected DogFACS actions between different cephalic types, ear shapes, and breeds. Only jowl length was reported to have an effect on the production of one action unit (i.e. “Upper lip raiser”: AU110)^6^, which is, however, not one of the facial indicators identified as emotional indicator in the present study.

Although facial expressions have been discussed as promising indicators of emotional states, it is important to consider them as part of only one of the components in the multifaceted response associated with emotional reactions^6^. Not only is it necessary to consider other components, such as behavioural tendencies and arousal levels, but also other modalities of emotional communication such as vocalizations, body expressions, and olfactory cues. Nonetheless our results demonstrate how close attention to facial detail can be used to help differentiate emotional states of different valence within a single carefully controlled context.

## Methods

### Ethical consideration

The experiment was approved by the College of Science Research Ethics Committee, University of Lincoln (UK) (UID CoSREC304) and complies with the “Guidelines for the Treatment of Animals in Behavioural Research and Teaching” of the Association for the Study of Animal Behavior (ASAB).

### Subjects

To reduce effects of morphological variation, 29 subjects of one breed without extreme facial features (Labrador Retriever) were tested (19 females – 13 neutered, 10 males – 9 neutered; age range: 2–9.5 years, mean age = 5.22 years). The sample size was determined based on two previous studies investigating dogs’ facial expressions using DogFACS (^26^: N = 29, ^44^: N = 24). Owners gave their written informed consent prior to the study.

### Experimental set-up

**Figure 2:**
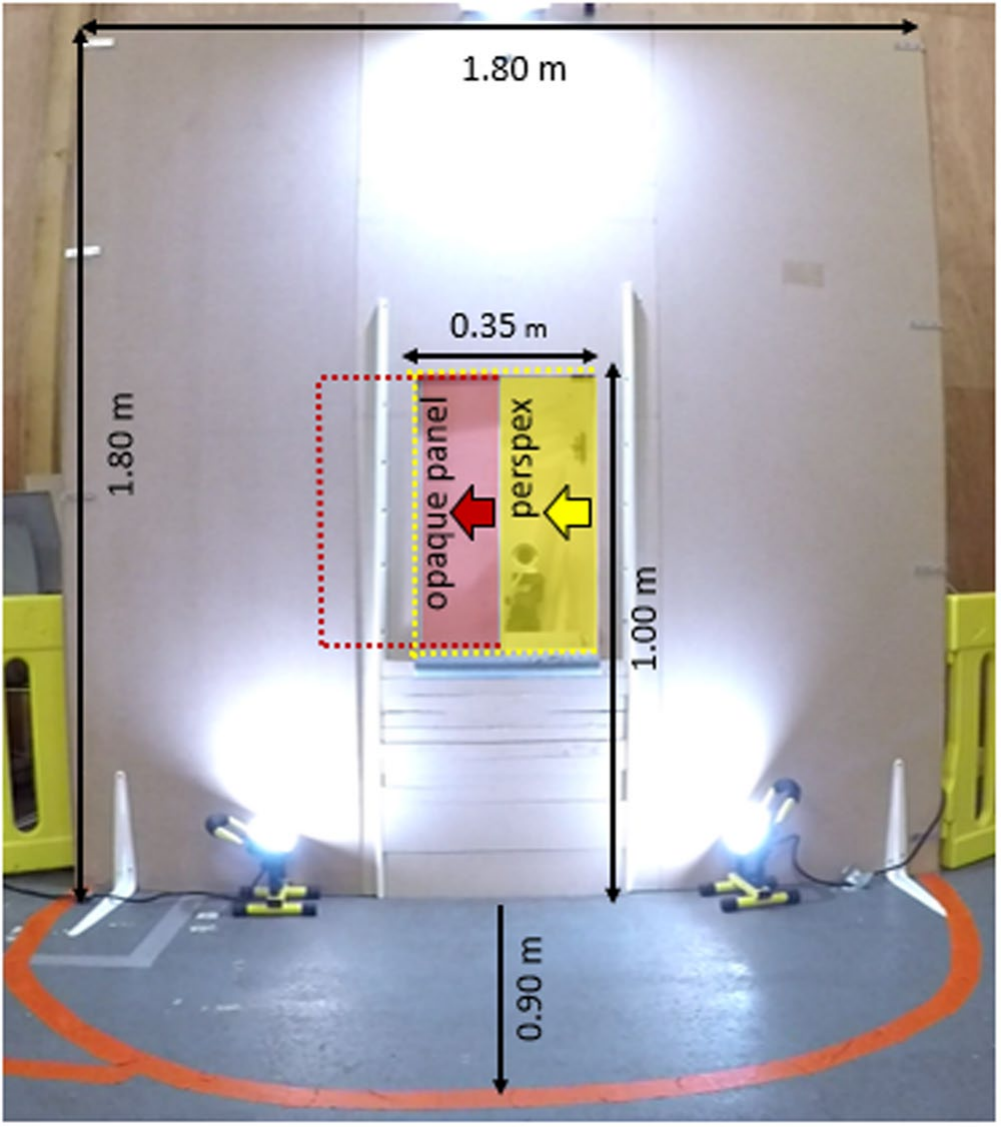
Experimental apparatus and goal area with measures. For improved visibility, the opaque panel (red) and the perspex (yellow) were coloured for this figure.

**Figure 3:**
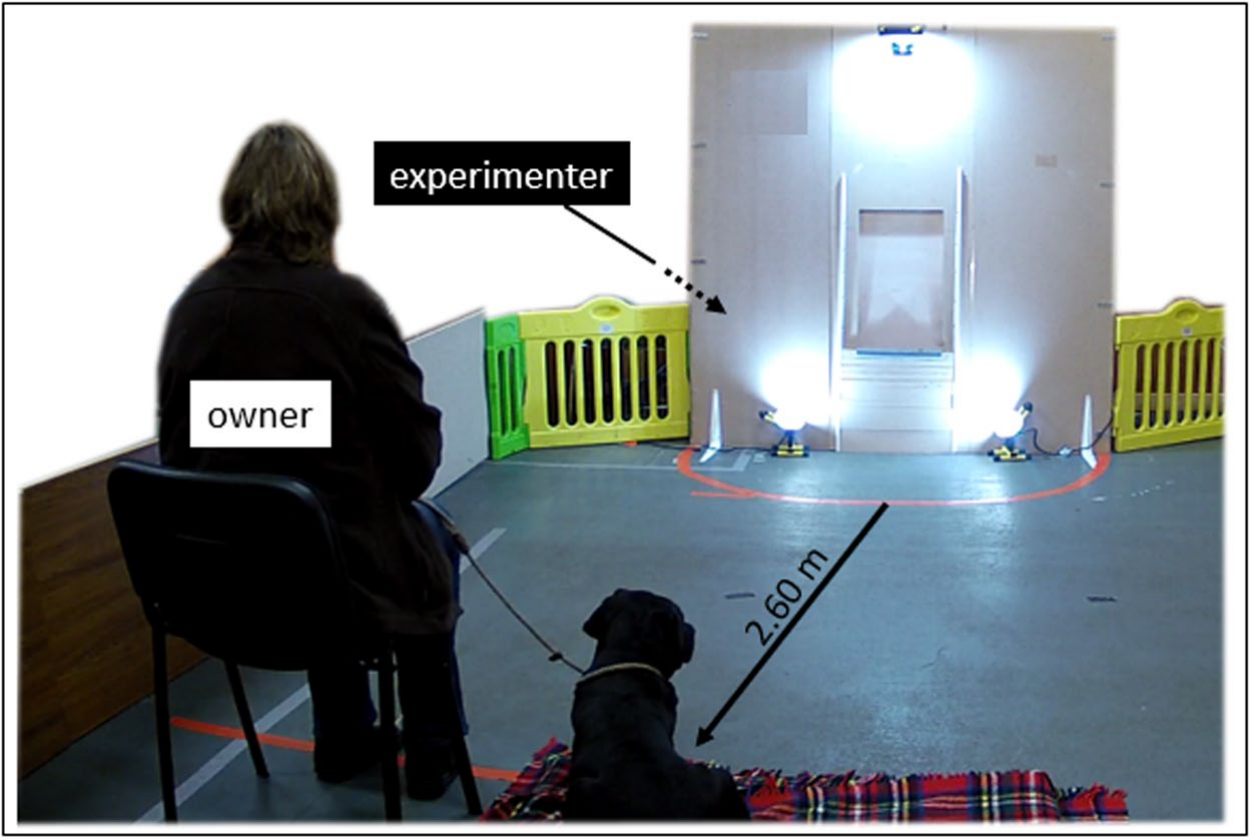
Experimental arena with dog and owner in the starting position. The experimenter remained hidden behind the wooden wall throughout the experiment.

The dogs were tested in a room measuring 7.50 × 4.00 m at the Riseholme Campus of the University of Lincoln (UK). The experimental set-up (Figs. 2 and 3) consisted of a 1.80 × 1.80 m wide wooden wall with an opening in the middle (50 cm from the floor), leading to a goal box attached on the reverse side of the wall. The experimenter, sitting to the left and out of sight of the dog behind the wooden wall, placed the food into the goal box. A removable transparent perspex panel prevented the dogs from accessing the item in the goal box straight away. When the experimenter slid the perspex sideways, the dog could access the item placed inside the goal box. To block the dog’s view of the goal box between trials, a movable opaque panel was used. A semicircle with a radius of 0.90 m was marked on the floor in front of the wooden wall (subsequently called “goal area”), which was a relevant measure of distance between the dog and the goal box. The dog’s (and owner’s) starting point was 2.60 m from the wooden wall (Fig. 3). The owner was sitting on a chair, wearing sunglasses to prevent inadvertent cueing. Owners were instructed to ignore the dog except when they were signalled to put on or remove the leash. A camera in the goal box was used to record dogs’ facial expressions during the experiment (camera: HIKVision, IR Mini Bullet Network Camera. Recorder: HIKVision, DS-7600 Series).

#### Experimental design

The experiment was separated into four steps: (1) Baseline, (2) Training I - Establishment of food anticipation, (3) Training II - Consolidation of food anticipation, (4) Testing. Each step was conducted in a separate experimental session with an inter-session interval of at least one day (on average 9.1 days).

### Step 1: Baseline

#### Habituation

Initially, owner, dog, and experimenter went into the experimental room and approached the open and empty goal box. The experimenter encouraged the dog to investigate the goal box and repeatedly opened and closed both panels to habituate the dog to the mechanism. Afterwards, dog and owner waited briefly in the adjacent room while the experimenter hid behind the apparatus, where she stayed throughout the experiment, invisible to the dog. The dog and the owner then re-entered the experimental room. The owner sat down on the chair and removed the dog’s leash and collar; the dog was allowed to freely move around until it showed signs of relaxation (e.g. calmly exploring the room or interacting with the owner, taking up a resting position, etc.) or after a maximum of 5 min. After the habituation phase, the Baseline session commenced.

#### Baseline session

Originally, we aimed to collect baseline measures of each dog’s facial expressions in a situation in which the individuals showed interest but without a strong emotional connotation. For this aim, a habituation-dishabituation procedure was used with novel objects (different assortments of duplo bricks, assumed to be of neutral valence to the dogs) presented in the goal box. The majority of dogs, however, showed signs of high arousal and possibly distress in this context (e.g. jumping at the owner, biting into the leash, avoiding to approach the goal box, etc.), and they did not face the goal box long enough to sample video clips for the subsequent video analysis. Therefore, this session was stopped and we only analysed positive and negative trials of the Testing session (Step 4) for every subject.

### Step 2: Training I - Establishment of food anticipation

Dogs were trained in repeated trials of the positive condition (subsequently labelled as “positive trials”) to expect a high-quality food reward (one piece each of a slice of boiled chicken and sausage) in the goal box. Motivation to consume the food was tested at the beginning of the session outside the experimental room by giving the dog one piece of both food types to eat. As in the Baseline session, the owner entered the experimental room with the leashed dog and sat down on the chair while the experimenter stayed behind the apparatus. Each trial started with a pre-trial phase (i.e. inter-trial interval; duration: 30 s) in which the dog was on leash near the owner and the opening of the goal box was blocked by the opaque panel. After 30 s, the experimenter signalled the beginning of the trial to the owner via a visual cue. In trials 1–5, in response to this cue, the owner walked the leashed dog to the margin of the goal area, removed the leash and collar, and gave a verbal and visual (hand signal) release cue. Afterwards, the owner remained standing and ignored the dog. From trial 6 on, in response to the experimenter’s cue, the owner remained sitting and immediately unleashed the dog, followed by the visual and verbal release cue. This allowed us to assess whether the dogs approached the goal area on their own, providing information about their state of training regarding the association of the goal box with the expected food reward as well as about their motivation to obtain the reward in the goal box.

In each trial, as soon as the dog entered the goal area with at least one forepaw, the opaque panel was removed (i.e. start of the anticipation phase), but the perspex still blocked the opening. After 5 s, the experimenter placed the food reward into the goal box using a long spoon in order to reduce visibility of a human body part. Immediately afterwards, the experimenter (still invisible for the dog) slid the perspex sideways from behind the wooden wall. Then the dog could access and eat the reward. The anticipation phase was set at 5 s as it allowed us to record the dogs’ facial expressions over several seconds while keeping the latency until the reward could be accessed short to avoid frustration. After the dog had consumed the reward, both panels were repositioned again so that the opening was blocked. A visual cue by the experimenter indicated to the owner to leash the dog and, in trials 1–5, to walk back to the chair. If the dog did not enter the goal area within 60 s after the release command (i.e. “no-response”), the experimenter signalled the owner to leash the dog and a new trial commenced. A “no-response” never occurred in trials 1–5 with any dog, as dogs were guided on lead to the goal area by the owner. From trial 6 onwards, only one dog showed a “no-response” in four trials.

In order to proceed to experimental Step 3, dogs were required to reach a predetermined learning criterion. Therefore, we evaluated the dog’s response to the release cue from trial 6 onwards where the owner remained sitting on the chair and the dog could approach the goal area alone. The criterion was that in five consecutive trials, the dog approached the goal area immediately after the release cue and remained focused on the goal box until the food reward was dispensed. Based on this criterion, the number of trials each dog received in this step was variable (mean number of trials = 14.20). The minimal number of trials to reach the predetermined learning criterion was 10, i.e. the five trials (trial 1–5) in which the owner was accompanying the dog to the goal area (as described above, for these trials the dog’s response to the release cue was not evaluated) and the five subsequent evaluated trials (trial 6–10) in which the dog could approach the goal area on its own. Six dogs that required more than the minimal amount of training trials still reached the required learning criterion in the first training session. The maximum number of trials per training session was 20. If a dog did not reach the required criterion by this time, another Step 2 session was repeated on a different day, following the same procedure. This was required for four subjects (the inter-session interval between these two training sessions was at least one day, and on average 12.5 days).

### Step 3: Training II - Consolidation of food anticipation

In this session, 20 positive trials were conducted using the same procedure as from trial 6 onwards in Step 2, with the owner remaining sitting on the chair.

### Step 4: Testing

In the last session, 15 positive trials were conducted using the same procedure as in the previous session. Additionally, the dogs were intermittently confronted with five trials of the negative condition in which access to the food reward was blocked (subsequently labelled “negative trials”).

This session always started with five positive trials. The following 15 trials included the five negative trials randomly interspersed (i.e. 75% positive and 25% negative trials overall). Once in this session two negative trials were scheduled to occur consecutively; but exactly when this occurred in the session was random.

In the negative trials, the opaque panel was removed when the dog entered the goal area with at least one forepaw and the food reward was placed into the goal box 5 s later, as in the positive trial. However, the perspex was not removed; thus, the dogs were able to see, but not access the food (i.e. start of the frustration phase). The negative trial was ended when the dog had left the goal area and did not re-enter for 15 s, or 55 s after the food was put into the goal box if the dog did not leave the goal area for more than 15 s. When the dog did not approach the goal area at all during a positive or negative trial (i.e. “no-response”), this trial was ended 60 s after the release command. The opaque panel was then returned and the experimenter signalled the owner to leash the dog again. This occurred with four dogs in 15 positive trials overall (of which 8 were directly following a negative trial) and with three dogs in three negative trials overall.

### Video sample preparation

**Figure 4:**
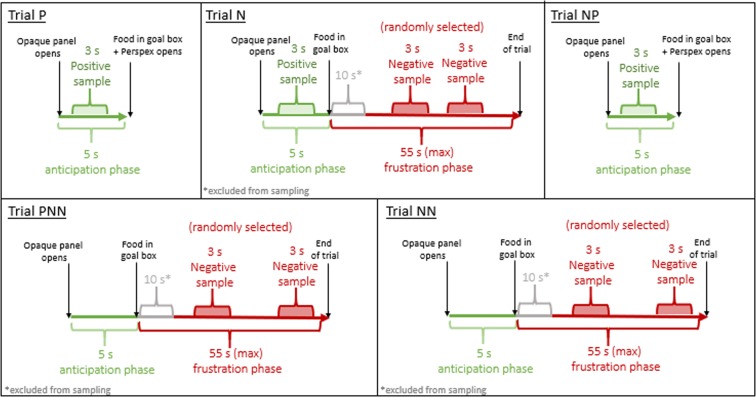
The three anticipation and three frustration phases of the five predetermined Testing trials (P, N, NP, PNN, NN) with time intervals used for the video sample preparation for the subsequent DogFACS coding.

For the subsequent DogFACS coding, video samples of 3 s length each were cut using Avidemux software (version 2.6.1). For each subject, video samples were prepared using three anticipation and three frustration phases from five pre-determined Testing trials (Step 4): the positive trial preceding the first negative trial (labelled “P”); the first negative trial (labelled “N”); the positive trial following the first negative trial (labelled “NP”); the first and second of the two consecutive negative trials (labelled “PNN” and “NN”, respectively). From the anticipation phase of trials P, N, and NP, one positive sample each was cut (Fig. 4). From the frustration phase of trials N, PNN and NN, two negative samples each were cut due to the longer duration of the negative compared to the positive trial (Fig. 4).

General eligibility criteria were that for the duration of the sample, dogs must be within the goal area with at least one forepaw and the face must be visible with the goal box camera for at least 2 s. Positive samples started 1 s after the onset of the anticipation phase to minimise possible distractions such as the movement of the panels. For each negative sample, the starting point within the 55 s (max.) frustration phase was randomly selected (using the R random number generator, function ‘sample’, repetitions excluded) within the limit that the first 10 s of each frustration phase were excluded, as the onset of frustration might not immediately occur. If the general eligibility criteria were not met for the duration of the negative sample, another random number was generated. Overall, 248 video samples meeting the eligibility criteria were prepared (for 13 samples the eligibility criteria were not met).

### Outcome measures

**Figure 5:**
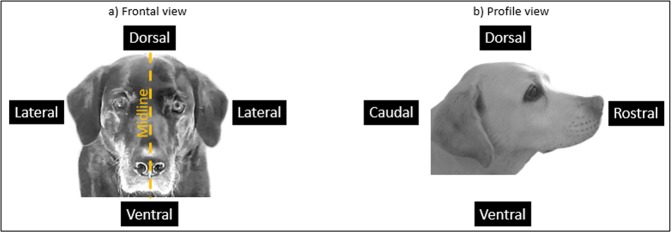
Directional terms used for the definition of the DogFACS^26^ variables in Table 4.

The video samples were coded based on the DogFACS manual^26^ (www.dogfacs.com) by a certified DogFACS coder (N.A.S.). All Upper and Lower Face Action Units (Action Unit = AU, i.e. muscular basis of the movement is known), all Action Descriptors (Action Descriptor = AD, i.e. muscular basis of the movement is not known), all Ear Action Descriptors (Ear Action Descriptor = EAD), and “Panting” as AD from the Gross Behaviour Codes were coded as present or absent in the 3 s samples (see Table 4 for an overview of the relevant DogFACS variables and Fig. 5 for a description of relevant anatomical directional terms). Videos were randomly renamed and the order of video samples was randomized (using the software Ant Renamer version 2.12) so that the coder was blind to the condition. The neutral ear position of our target breed was determined by collecting images of sleeping Labradors, as suggested in the DogFACS^26^ manual, as well from four subjects in the baseline session of this experiment that did not show any arousal behaviours (e.g. tail wagging, panting, lip licking) for 2 s before and 2 s after the second in which the image was taken. Coding was performed using Solomon Coder (version 15.03.15, Andràs Péter). Only DogFACS^26^ variables with a prevalence above 10% in at least one of either all positive or all negative video samples were used for subsequent analysis (see Table 4 for the variables meeting this criterion). We assessed the prevalence separately for the positive and negative trials to avoid excluding variables that primarily occurred only in one of both conditions.

**Table 4:** DogFACS^26^ variables used as outcome measures in this study (AU = Action Unit; AD = Action Descriptor; EAD = Ear Action Descriptor).

Category	AU/AD/EAD	Number	Variable name	Definition*
Upper Face Action Units	AU	101	**Inner brow raiser**	Protuberance above the eye moves dorsally and obliquely towards the midline.
		143	Eye closure	Both eyelids move towards and touch each other, covering the eye for at least 0.5 s.
		145	**Blink**	Both eyelids move towards and touch each other, covering the eye for less than 0.5 s.
Lower Face Action Units		109 + 110	(Nose wrinkler & Upper lip raiser)**	Nose and upper lip move dorsally and wrinkles appear on the dorsal muzzle part.
		110	(Upper lip raiser)**	Upper lip moves dorsally.
		12	**Lip corner puller**	Lip corners move caudally.
		116	**Lower lip depressor**	Lower lip moves ventrally.
		118	Lip pucker	Lip corners move rostrally.
		25	**Lips part**	Any lip separation.
		26	**Jaw drop**	Lower jaw moves ventrally in a relaxed manner (i.e. absence of tension signs) and teeth are separated.
		27	Mouth stretch	Lower jaw moves ventrally in an actively stretching manner and teeth are separated; lower teeth, tongue and oral cavity are visible.
Action Descriptors	AD	19	**Tongue show**	Tongue is protruded at least until the inner lower lip.
		33	Blow	Lips expand due to air being expelled from the mouth.
		35	Suck	Upper lip is sucked into the mouth.
		37	Lip wipe	Tongue moves on the outer part of the upper lips from the midline of the mouth to one corner.
		137	**Nose lick**	Tongue moves out of the mouth towards the nose and wipes it.
Gross Behaviour		126	**Panting**	Mouth is open (AU26), tongue is protruded (AD19), and dog breathes shortly and quickly.
Ear Action Descriptors	EAD	101	(Ears forward)**	Ears move rostrally.
		102	**Ears adductor**	Ears move dorsally towards the midline of the head; bases of both ears come closer together.
		103	**Ears flattener**	Ears move caudally.
		104	Ears rotator	Ears move laterally and externally.
		105	Ears downward	Ears move ventrally.

DogFACS^26^ variables with bold letters occurred with a prevalence of at least 10% in either all positive or all negative samples. *Definitions were obtained from the DogFACS manual^26^ (www.dogfacs.com) and were partly adapted for this Table. **DogFACS^26^ variables in brackets were excluded from the analysis as the strength of the intercoder reliability was below a substantial agreement^61^.

### Analyses

#### Intercoder reliability

Intercoder reliability analysis was performed in RStudio 1.0.153 (function: cohen.kappa; package: psych^92^). For 25 randomly selected samples (>10% of all samples, selected by using the R random number generator, function ‘sample’, repetitions excluded), all DogFACS^26^ variables with a prevalence above 10% in either all positive or all negative samples were coded by a second certified DogFACS^26^ coder (A.B.). Except for three variables (“Nose wrinkler & Upper lip raiser”: AU109 + 110; “Upper lip raiser”: AU110; “Ears forward”: EAD101), all variables had at least a substantial strength of intercoder agreement^61^ (Supplementary Table 1). Accordingly, eleven variables (“Inner brow raiser”: AU101; “Blink”: AU145; “Lip corner puller”: AU12; “Lower lip depressor”: AU116; “Lips part”: AU25; “Jaw drop”: AU26; “Tongue show”: AD19; “Nose lick”: AD137; “Ears adductor”: EAD102; “Ears flattener”: EAD103; “Panting”: AD126) were used for the final analyses (Tables 2 and 3).

#### Statistical analyses

Statistical analyses were performed in RStudio 1.0.153. Our first aim was to assess whether there were any differences in facial expressions in repeated samples of the same condition (i.e. all positive samples or all negative samples, respectively), with the preceding trial being either of the same or the other condition. For this aim, binomial mixed effect models were calculated (function: glmer; package: lme4^93^) separately for the data of all coded positive and negative samples. The eleven selected DogFACS variables (see Tables 2 and 3) were used as response variables, sample was used as a predictor variable (samples of the positive condition were from the anticipation phase of trials P, N, NP. Samples of the negative condition included the two each from trials N, PNN, NN), and subject ID was used as a random effect. As there was no indication that inclusion of sample as a predictor variable added significant information to explaining our data, we pooled the data of all positive and of all negative samples, respectively. In order to assess which facial expressions differed between the positive and negative condition, we calculated binomial mixed effect models with the eleven DogFACS variables as response variables, condition (positive/ negative) as a predictor variable and subject ID as a random effect. Subject sex and age were used as blocking factors. To control for type-I-errors, the Holm-Bonferroni method was applied to correct for multiple hypotheses testing.

## Supplementary information

Supplementary information

## Data Availability

The dataset generated and analysed during the current study is available from the corresponding author on reasonable request.
