# Supplementary material for: Differences in facial expressions during positive anticipation and frustration in dogs awaiting a reward
Source: Sci Rep. 2019 Dec 17;9:19312. doi: 10.1038/s41598-019-55714-6 (PMC6917793; doi:10.1038/s41598-019-55714-6)
Supplement: Supplementary file 1 — Supplementary information [file 41598_2019_55714_MOESM1_ESM.pdf]

Differences in facial expressions during positive anticipation and frustration in dogs awaiting a reward

Annika Bremhorst[1,2,3,*], Nicole A. Sutter[1], Hanno Würbel[1], Daniel S. Mills[3], and Stefanie Riemer[1]

[1] Division of Animal Welfare, DCR-VPHI, Vetsuisse Faculty, University of Bern, 3012 Bern, Switzerland

[2] Graduate School for Cellular and Biomedical Sciences (GCB), University of Bern, 3012 Bern, Switzerland

[3] School of Life Sciences, University of Lincoln, Lincoln LN6 7DL, United Kingdom

* annika@bremhorst.de

Supplementary information:

Supplementary Table 1. Results of the intercoder reliability assessment.

DogsFACS variable	Cohen's Kappa
Inner brow raiser (AU101)	0.75
Blink (AU145)	0.8
Nose wrinkler and upper lip raiser (AU109+110)	0.5
Upper lip raiser (AU110)	0.19
Lip corner puller (AU12)	1
Lower lip depressor (AU116)	1
Lips part (AU25)	1
Jaw drop (AU26)	0.9
Tongue show (AD19)	1
Nose lick (AD137)	1
Ears forward (EAD101)	0.52
Ears adductor (EAD102)	0.78
Ears flattener (EAD103)	0.92
Panting (AD126)	1
